# T Cell Surveillance during Cutaneous Viral Infections

**DOI:** 10.3390/v16050679

**Published:** 2024-04-26

**Authors:** Luxin Pei, Heather D. Hickman

**Affiliations:** Laboratory of Viral Diseases, National Institute of Allergy and Infectious Diseases, National Institutes of Health, Bethesda, MD 20892, USA; luxin.pei@nih.gov

**Keywords:** antiviral T cells, cutaneous microenvironment, poxvirus, herpes simplex virus

## Abstract

The skin is a complex tissue that provides a strong physical barrier against invading pathogens. Despite this, many viruses can access the skin and successfully replicate in either the epidermal keratinocytes or dermal immune cells. In this review, we provide an overview of the antiviral T cell biology responding to cutaneous viral infections and how these responses differ depending on the cellular targets of infection. Much of our mechanistic understanding of T cell surveillance of cutaneous infection has been gained from murine models of poxvirus and herpesvirus infection. However, we also discuss other viral infections, including flaviviruses and papillomaviruses, in which the cutaneous T cell response has been less extensively studied. In addition to the mechanisms of successful T cell control of cutaneous viral infection, we highlight knowledge gaps and future directions with possible impact on human health.

## 1. Introduction

The skin is the largest organ in the body that forms an extensive physical barrier against invading pathogens. The anatomical composition of the skin establishes a unique niche to harbor viral replication and provides a scaffold for antiviral immune responses [1]. As a stratified tissue, the skin has three main layers: epidermis, dermis, and subcutaneous adipose tissue. Specialized structures such as hair follicles, sebaceous glands, sweat glands, and nerves are also embedded in the cutaneous layers. The epidermis forms the outermost layer of the skin and is comprised predominantly of keratinocytes. Basal keratinocytes, located closest to the dermis, slowly proliferate at homeostasis to maintain skin integrity. After keratinocytes first move to the suprabasal epidermal layers, they gradually differentiate while continuing to move to the outer epidermal layers. Terminally differentiated and dead keratinocytes accumulate in the outermost layer of the epidermis, termed the stratum corneum, which forms a water-resistant physical barrier. Dead cells are also shed from the skin in a process called desquamation. Keratinocyte maturation is tightly regulated to maintain the structural integrity of the skin.

Beneath the epidermis lies the collagen- and elastin-rich dermis. Collagen provides the skin’s mechanical and structural integrity, while elastin imparts the skin with significant plasticity. Undulations in the epidermis called Rete ridges form at the epidermal–dermal junction, anchoring the two layers together and contributing to mechanical strength and skin homeostasis [2]. Blood and lymph vessels form a dense, interwoven network throughout the dermis. During inflammation or infection, changes in the blood vasculature promote the recruitment of immune cells. Lymphatic vessels collect fluid from the interstitial tissue space, routing fluid unidirectionally to the draining lymph node [3]. Lymphatic vessels have long been appreciated as “super-highways” for dendritic cells (DCs) to traffic rapidly from the skin to the draining lymph node rather than crawling through densely packed tissue [4,5]. Recent studies also show that T cells can also use lymph vessels to egress from the skin and reach the draining node [6].

**Figure 1:**
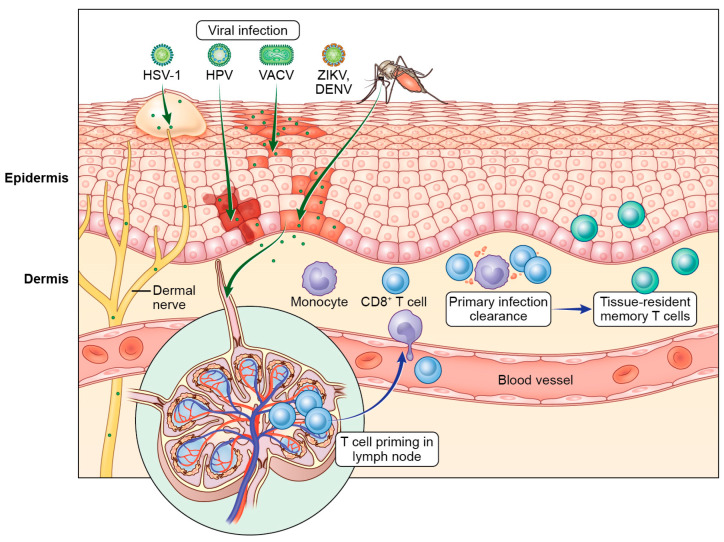
T cell control of cutaneous viral infections. Viruses such as vaccinia virus (VACV), herpes simplex virus type 1 (HSV-1), Zika virus (ZIKV), dengue virus (DENV), and human papillomavirus (HPV) breach the skin barrier to establish infection in the epidermis and dermal immune cells. T cells are primed and activated in the draining lymph node by antigen-presenting cells (APCs) and then enter circulation. From the blood, activated effector T cells migrate into infected tissues. In the skin, cytotoxic T cells target and kill infected cells to limit pathology. Our knowledge of cytotoxic elimination in the skin is limited for some viruses, such as HPV. A small proportion of T cells will remain in the skin and differentiate into tissue-resident memory T cells (T_RM_) that can offer rapid responses to reinfections.

In addition to its physical properties, the skin is protected by a complex immune network, even at steady state. Antigen-presenting cells (APCs), including Langerhans cells (LCs) and DCs, and tissue-resident T cells reside in the epidermis where they slowly patrol the barrier. When viruses overcome neutralization or inactivation by the humoral and innate immune response to establish infection, cytotoxic T cells are then critical for eliminating infected cells. After primary infection is cleared, T cells can establish long-term residency in the skin that protects the host from homologous viral infections [7]. This review discusses seminal findings and recent discoveries regarding T cell biology during cutaneous viral infections, topically divided by virus. We compare T cell responses in characterized mouse models of poxvirus and herpes simplex virus infection, followed by recent data extending to vector-borne flaviviruses and papillomaviruses (Figure 1). We further explore the cellular spatial organization and the impact of the local microenvironment on T cell responses to cutaneous viral infection. Together, these studies yield a broader understanding of a vital cell subset that rapidly and potently responds to viral infections of a critical barrier tissue.

## 2. Poxviruses

### 2.1. Cutaneous Vaccinia Virus Infection

Vaccinia virus (VACV) is a large double-stranded DNA virus in the *Orthopoxvirus* genus. In the late 1900s, the epicutaneous inoculation of VACV led to the eradication of deadly human smallpox infections caused by a related poxvirus, variola virus (VARV) [8,9]. Due to conserved viral proteins, VACV vaccination successfully elicits a cross-reactive immune response that confers long-term protection against VARV [10]. VACV immunization induces polyfunctional CD8^+^ T cell responses that can produce high levels of interferon (IFN)-γ in healthy participants, offering protection against reinfection [11]. VACV vaccination also elicits a long-lived adaptive immune response, with VACV-specific antibodies stable for 75 years and antigen-specific T cells in the peripheral blood exhibiting a half-life of 8–15 years [10]. The longevity of VACV-specific tissue-resident memory T cells in human skin is currently unknown. In humans, both the humoral and cell-mediated immune response are thought to contribute to protection based on enhanced infection and complications experienced by immunocompromised individuals who were vaccinated during the smallpox eradication campaign [12,13].

**Figure 2:**
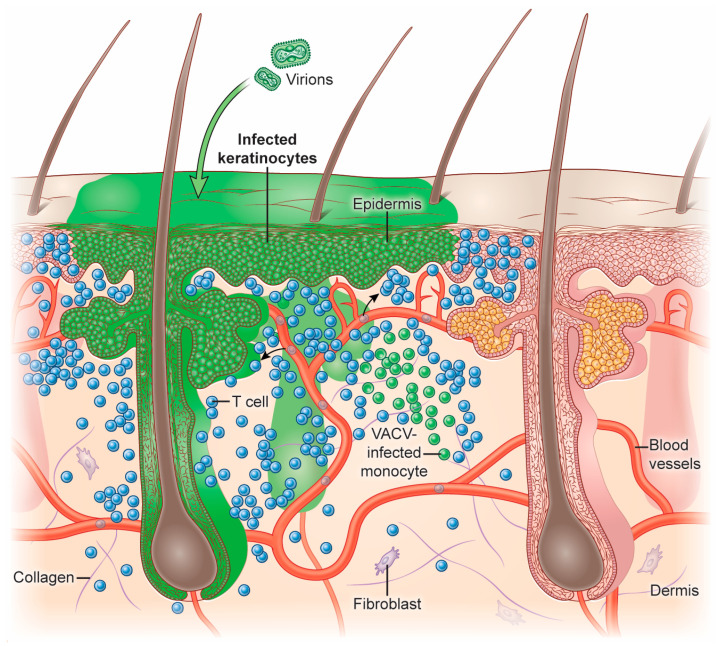
VACV infection of the skin. After VACV inoculation into the mouse skin using a bifurcated needle, the virus infects non-hematopoietic and immune cells. VACV (shown here in green) replicates in epidermal keratinocytes and preferentially infects hair follicle cells. Monocytes recruited to the infected skin also become infected. CD8^+^ T cells can eliminate VACV-infected monocytes in the skin.

VACV can infect many cells and tissues in mice, including the skin, lungs, brain, and peritoneum. The inoculation of VACV into mice using epicutaneous scarification or intradermal injection has been used extensively for T cell studies [14,15,16,17,18,19]. After inoculation into the skin, VACV replicates in epidermal keratinocytes, inducing their proliferation and migration [16,20,21]. In the dermis, VACV can infect recruited leukocytes, including DCs, monocytes, neutrophils, and macrophages [16,22,23] (Figure 2).

### 2.2. T Cell Activation during VACV Infection

After VACV establishes infection in the skin, CD8^+^ T cells become primed and activated in the draining lymph node (dLN) proximal to the site of infection [15,24,25]. The lymph nodes are highly organized secondary lymphoid organs. Their structure facilitates and optimizes the ability of recirculating naïve T cells to find APCs carrying cognate antigen [26]. After subcutaneous VACV injection into the skin, both DCs and macrophages located in LN sinuses capture lymph-borne VACV virions and become infected [25,27]. Alternatively, at high viral doses, a few virions can enter the LN conduit system to infect DCs located in the center of the LN [27]. Both DCs and macrophages can process viral proteins synthesized within the infected cell or internalized viral proteins. APCs can then present viral peptides complexed with MHC class I molecules through direct or cross-presentation [28,29]. Naïve T cells enter the LN through centrally located high endothelial venules, where they can encounter APCs [30,31]. Chemokines produced by LN DCs, including CCL3, CCL4, and CCL5, can facilitate CD8^+^ T cell/DC interactions and T cell priming during VACV infection [29]. During the later stages of LN infection, XCR1^+^ DCs are critical for CD8^+^ T cell priming [32]. After priming and activation in the dLN, CD8^+^ T cells alter receptor expression, including the downregulation of cell-surface L-selectin [33]. Activated T cells then exit the node, re-enter the blood, and traffic to the site of infection.

### 2.3. T Cell Recruitment to the Skin and Cytotoxic Activity

VACV infection results in inflammation that promotes dramatic changes in the local vascular and lymphatic endothelium [34]. Activation of vascular endothelial cells allows leukocytes to adhere to the endothelium and transmigrate through the vessels and into the tissue. Activated CD8^+^ T cells express glycosylated P-selectin glycoprotein ligand-1 (PSGL-1) termed cutaneous lymphocyte antigen (CLA), CD43, and CD44 to bind to the endothelial adhesion molecules P/E-selectin [35,36,37,38]. Blockade or genetic knockout of adhesion molecules can lead to impaired T cell entry to the site of infection [39].

VACV skin infection has been successfully visualized using multiphoton microscopy (MPM) to characterize T cell movement and function in live mice [40]. As effector CD8^+^ T cells exit the vasculature near areas of VACV infection, the skin microenvironment significantly impacts the location of CD8^+^ T cells. Even though VACV primarily replicates in epidermal keratinocytes, antigen-specific CD8^+^ T cells do not effectively infiltrate the epidermis to clear these lesions [16]. Using MPM, cytotoxic CD8^+^ T cells have been observed directly lysing VACV-infected monocytes in the dermis [16,41]. During this process, chemokines are essential determinants of T cell location and effector function. After VACV infection, the chemokines CXCL9 and CXCL10 are considerably upregulated in infected skin [41]. Activated CD8^+^ T cells can express CXCR3, the receptor for CXCL9 and CXCL10 [42]. Mice genetically lacking CXCR3 have a significantly reduced ability to prevent VACV spread in the skin, specifically in both keratinocytes and monocytes [41]. Mechanistically, *Cxcr3*^−/−^ VACV-specific T cells cannot effectively enter areas containing high numbers of infected monocytes, decreasing T cells’ ability to find infected cells.

Cytokine production also contributes to T cell-mediated clearance of VACV-infected skin. Paradoxically, many VACV-specific T cells co-produce the anti-inflammatory cytokine IL-10 and the potent antiviral cytokine IFN-γ [43]. Importantly, T helper 1 (Th1) cells have been shown to switch from an inflammatory IFN-γ-producing cell to an IL-10-producing cell during differentiation, which is essential to limit T cell-driven immunopathology [44]. Using MPM imaging and IL-10 reporter mice, Cush et al. demonstrated that IL-10-producing T cells accumulate near VACV-infected cells or in areas adjacent to viral lesions [43]. Despite these studies, much remains to be learned about the precise mechanisms of CD8^+^ T cell-mediated viral clearance in vivo, including its regulation and participation in later stages of the response during tissue healing.

To better understand T cell immunodominance during VACV infection, Tscharke et al. mapped VACV epitopes in both C57BL/6 and Balb/C mice [17,45]. Although CD8^+^ T cells responded to a broad number of peptides from an assortment of viral proteins, the most dominant response after intraperitoneal infection of C57BL/6 mice was against a peptide from the secreted interferon-gamma decoy receptor B8R [46]. Intradermal infection focused the CD8^+^ T cell response to B8R, with almost all T cells in the skin responding to this determinant [17]. 

In addition to CD8^+^ T cells, CD4^+^ T cells are also important for controlling VACV infection; however, results differ based on the route of viral inoculation. Moutaftsi et al. demonstrated that VACV-specific CD4^+^ T cells recognize determinants from different viral proteins compared to CD8^+^ T cells, including viral structural and regulatory proteins expressed at later stages of VACV infection [47,48]. These findings are consistent with a role for cross-presentation during CD4^+^ T cell activation after the transfer of late VACV proteins to APCs [49]. Cytotoxic responses and robust IFN-γ production by CD4^+^ T cells have also been observed after intraperitoneal VACV infection [50]. During this infection, either the depletion of CD4^+^ T cells or decreased MHC class II expression reduced protection [50,51]. Most recently, effector memory Th1 cells have been shown to be effective in protecting against VACV skin infection [52]. Evidence also exists for the importance of CD4^+^ T cells during the anti-VACV response in humans. During acute human infection, CD4^+^ T cells upregulate the activation and degranulation markers CD38, CD45RO, and CD107a. Furthermore, activated CD4^+^ T cells increase the gene expression of cytolytic proteins, including granzymes and perforin [53].

### 2.4. Tissue-Resident T Cells during VACV Infection

After the clearance of primary VACV infection, a small number of T cells will persist in the tissue to become tissue-resident memory T cells (T_RM_), providing rapid protection against secondary infection [7,54]. The mechanisms regulating T_RM_ formation in different tissues have been a topic of intense investigation, and cutaneous VACV infection has been applied to study this question. Following VACV skin infection, a population of differentiated T_RM_ CD8^+^ T cells can be found in the skin, expressing the tissue residency markers CD69 and CD103 [55]. While cognate antigen is not needed for the trafficking of activated CD8^+^ T cells into the skin after VACV infection, T_RM_ longevity depends on local cognate antigen [55,56]. Recently, Abdelbary et al. reported that the T cell receptor (TCR)-signaling strength during VACV infection promotes T_RM_ CD8^+^ T cell residency in the skin [57]. The authors infected mice with an ensemble of recombinant VACV expressing peptide variants of SIINFEKL, which OT-I TCR-transgenic CD8^+^ T cells recognize with different affinity [58]. IFN-γ production by effector CD8^+^ T cells and the subsequent number of CD8^+^ T_RM_ established strictly correlate with TCR affinity [57].

In recent years, studies have also highlighted the importance of cellular metabolism on effector T cell and T_RM_ responses. VACV skin infection induces profound metabolic changes that reflect the contribution of both viral replication and recruited immune cells [59]. T cells have additional energy requirements during antiviral responses, drastically altering their cellular metabolism [60,61]. Interestingly, the formation of CD8^+^ T_RM_ in the skin critically depends on exogenous lipid uptake through fatty acid-binding proteins 4 and 5 [62]. Genetic deletion of these proteins or inhibition of mitochondrial fatty acid oxidation diminishes T_RM_ CD8^+^ T cell persistence.

Besides the development of T_RM_ from recruited TCRαβ effector T cells, the skin also possesses tissue-resident TCRγδ T cells that contribute to viral clearance, tissue maintenance, and wound healing [63]. Dendritic epidermal T cells (DETCs) express an invariant Vγ5 TCR [63]. These tissue-resident cells are seeded in the murine skin during fetal development and permanently reside in the epidermis as a relatively immobile immune population [63]. Dermal γδ T cells are composed of resident cells that originate from fetal development as well as γδ T cells recruited during infection [64]. Cutaneous VACV infection increases dermal γδ T cells by 10-fold due to cell infiltration rather than local expansion [64]. However, recruited dermal γδ T cells do not establish residency and exhibit differential expression of CD27, IL-17, and IFN-γ [65,66]. Specifically, recruited dermal CD27^+^ γδ T cells secrete granzyme B to control viral replication [64,67]. Recently, Lujan et al. explored the expression of a non-conventional granzyme, granzyme C, in skin-resident T cells [68]. DETCs, dermal γδ T cells, and CD8^+^ T_RM_ all expressed granzyme C at steady state. Following cutaneous VACV infection, granzyme C expression was significantly increased in DETCs and CD8^+^ T_RM_, suggesting that this serine protease might contribute to the antiviral response in the tissue.

Tissue-resident memory T cells offer a rapid immune response to viral reinfections. More studies are needed, perhaps using MPM imaging, to determine the mechanisms that allow memory T cells to locate areas of secondary infection and rapidly eliminate it before extensive viral spread.

## 3. Herpes Simplex Virus

### 3.1. Cutaneous Infection by HSV-1

Herpes simplex virus type 1 (HSV-1) is a highly prevalent human pathogen, with a global infection rate approaching 70% [69]. In immunocompetent individuals, HSV-1 infection is mild and self-limiting, often characterized by virus-induced vesicular lesions or cold sores on or near the lip. HSV-1 infection is also the cause of herpetic stromal keratitis, which can lead to corneal blindness [70]. Additionally, immunocompromised individuals and neonates can experience herpetic encephalitis, an often fatal infection of the brain [71,72].

HSV-1 is a double-stranded DNA virus with a primary lytic phase followed by a latent infection cycle. During the lytic phase, HSV-1 replicates in the epithelial cells of the skin, mucosa, and cornea before spreading along sensory neurons [73]. Latent HSV-1 can reside in the trigeminal ganglia for long periods before reactivation and disease recurrence [74]. During primary HSV-1 infection of the skin, neutrophils, monocytes, CD8^+^ T cells, and CD4^+^ T cells are recruited to control local viral replication [75,76,77,78].

T cell activation in response to HSV-1 infection has been heavily investigated and has led to the discovery of some of the paradigms in antiviral T cell priming. Naïve T cells are primed in the draining LN where DCs present HSV-1-derived viral antigens [79,80]. Before these studies, it was widely held that Langerhans cells located in the epidermis were the primary APCs that gathered viral antigen from the skin, migrated to the draining LN, and primed antiviral T cells. However, after cutaneous HSV-1 infection, the primary cells responsible for priming virus-specific T cells have been unequivocally shown to be CD8a^+^ DCs (now known as cDC1s), while Langerhans cells are dispensable [81]. A follow-up study showed that these DCs are generally essential for antiviral CD8^+^ T cell priming, including after VACV infection [82]. Later, this group also demonstrated that CD103^+^ DCs migrated from the skin and presented HSV-1 antigens to CD8^+^ T cells [83]. These studies, mainly performed after HSV-1 skin infection, solidified our understanding of cells in the LN that can activate virus-specific cytotoxic T cells after tissue infection.

Several studies have investigated the role of CD8^+^ T cells in clearing primary HSV-1 infection. Simmons and Tscharke elegantly demonstrated that antibody depletion of CD8^+^ T cells leads to increased HSV-1 spread and destruction of infected ganglionic neurons after cutaneous primary HSV-1 infection [84]. Another study found that CD8^+^ T cells are involved in the clearance of established lytic infection, but these cells cannot control viral spread [85]. In this study, the severity of skin infection was similar between transgenic mice lacking CD8^+^ T cells compared to wild-type animals. Conversely, CD4^+^ T cell-deficient animals present with increased susceptibility to cutaneous infection, highlighting the importance of CD4^+^ T cells during HSV-1 clearance [75].

With the advancement of technology, more recent studies have explored the interactions and spatial orientation of CD4^+^ and CD8^+^ T cells in HSV-1-infected skin at a higher resolution. Antigen-specific T cells produce IFN-γ during skin infection with HSV-1 [86]. Most IFN-γ-producing CD8^+^ T cells are localized in the epidermis and hair follicles. In contrast, IFN-γ-producing CD4^+^ T cells are more evenly distributed throughout the epidermis and dermis. Given the distinct localization of CD4^+^ and CD8^+^ T cells, CD4^+^ T cells can better interact with MHC-II-expressing DCs. In further support, antibody blockade of APC-costimulatory molecules has a limited effect on IFN-γ-producing CD8^+^ T cells. Lastly, the authors suggested that CD8^+^ T cells are likely to interact with HSV-1-infected cells in the epidermis, including keratinocytes and DETCs [86]. In a more recent study, genetic deletion of sensory neurons was shown to alter the spatial distribution of CD8^+^ T cells along with their response to cutaneous HSV-1 infection [78]. Ablation of sensory neurons induces immune cell infiltration to the infection site and increases inflammatory cytokine production (TNF and IL-1β), suggesting a role for the nervous system in regulating immune responses [78].

In summary, despite decades of investigation, delineating the exact mechanisms of CD8^+^ T cell-mediated clearance of HSV-1 lytic infection warrants further exploration. HSV-1 is a complex pathogen infecting both the epithelium and neurites in the skin before establishing latency. Understanding the specifics of such complicated viral clearance will undoubtedly illuminate other aspects of T cell biology.

### 3.2. Tissue-Resident Memory T Cells during HSV-1 Infection

After the clearance of primary HSV-1 infection, the virus establishes life-long latency in sensory neurons. Epidermal lesions can appear when the virus is reactivated after anterograde axon transport to the skin. HSV-1 reactivation can lead to the induction of virus-specific T_RM_, like VACV infection [87]. Despite a failure of HSV-1 to spontaneously reactivate in mice (unlike humans), HSV-1 infection has been used extensively to study T_RM_ development in the skin [87,88]. In mouse skin, CD4^+^ and CD8^+^ memory T cells are retained following clearance of primary HSV-1 infection. Interestingly, MPM imaging has revealed that CD4^+^ and CD8^+^ T_RM_ exhibit distinct localization in the skin and differences in motility during tissue surveillance [89]. CD4^+^ T_RM_ cells reside in both the epidermis and dermis and possess the capacity for rapid migration and re-entry into circulation. On the other hand, CD8^+^ T_RM_ are sequestered in the epidermis close to the original infection site. Due to their location in the tight confines of the epidermis, these cells possess low intrinsic motility and do not enter the vasculature to recirculate. CD8^+^ T_RM_ also display a dendritic-like morphology with long pseudopods. Interestingly, CD8^+^ T_RM_ compete with other skin-resident cells, such as DETCs, for tissue occupancy, displacing DETCs to reside in specific areas of the epidermis [90]. The extensive dendrites of CD8^+^ T_RM_ have been suggested to sample the environment and allow the rapid detection of viral antigens [91].

Several key cytokines have been shown to promote the development of CD103^+^ CD8^+^ T_RM_ cells in the skin after HSV-1 infection, including IL-15 and TGF-β [92,93]. IL-15 is produced in the skin by keratinocytes and Langerhans cells to maintain epidermal T_RM_, whereas TGF-β promotes CD103 expression and cell differentiation into less functionally malleable T_RM_ cells. Fonseca et al. examined transcriptional divergence between CD8^+^ and CD4^+^ T_RM_ in the skin, finding that the transcription factor Runx3 is critical for CD8^+^ T cell tissue residency but not for that of CD4^+^ T cells [94]. Interestingly, ectopic expression of Runx3 in CD4^+^ T cells renders them TGF-β responsive (like CD8^+^ T cells) and alters tissue distribution towards the skin epithelium. Park et al. later showed the development of CD8^+^ T_RM_ that produce IFN-γ (termed T_RM_1) is transcriptionally divergent from T_RM_ that produce IL-17 (T_RM_17) [95]. While HSV-1-induced T_RM_1 development depends on a T-bet-Hobit-IL-15 axis, T_RM_17 elicited by *Staphylococcus epidermidis* application develop independently from these factors. A more recent study also demonstrated that CD8^+^ T_RM_ develop from a killer cell lectin-like receptor G1 (KLRG1)-negative effector population accumulating in the skin after acute infection [96]. Thus, much of our knowledge about the requirements for the development of T_RM_ in the skin has been acquired during HSV-1 infection.

Less is known about the exact mechanisms that allow HSV-1-specific T_RM_ in the skin to prevent herpetic reactivation. After TCR stimulation, CD8^+^ T_RM_ upregulate the production of IFN-γ and cytolytic molecules such as granzyme B [97]. IFN-γ is critical for maintaining HSV-1 latency through remodeling the tissue transcriptome. *Ifng*^−/−^ T_RM_ induce a decreased tissue response compared to IFN-γ-producing T_RM_ [98]. Thus, although the tissue environment appears important for preventing HSV-1 spread, we still have much to learn about the direct interactions of T_RM_ with virus-infected cells in the skin.

## 4. Flaviviruses

### 4.1. Flavivirus Skin Infection

Insect bites circumvent the protective barrier of the skin against viral infection by depositing the virus directly into the epidermis and dermis. Arboviruses, such as Zika virus (ZIKV) and dengue virus (DENV) transmitted by the mosquito *Aedes aegypti*, continue to raise serious public health concerns due to explosive outbreaks (ZIKV) and high disease prevalence globally (DENV) [99,100]. Following cutaneous infection, both ZIKV and DENV disseminate from the skin to the draining LN and blood, causing systemic disease. After DENV infection, individuals may experience fever, arthralgia, myalgia, abdominal pain, and rash. In some cases, hemorrhagic fever/dengue shock syndrome may develop after the fever subsides or after a secondary infection with a different DENV serotype, which leads to increased mortality [101,102]. ZIKV infection in adults is usually asymptomatic and self-limiting, though fever and rash are also common. However, the ZIKV outbreak of 2015–2016 was characterized by congenital ZIKV syndromes, including microcephaly and cerebral malformations [103,104]. Compared to other skin-tropic viral infections, such as those with VACV or HSV-1, detailed knowledge of the cutaneous T cell response to DENV and ZIKV is lacking. This is partly driven by (1) the lack of small animal models that readily recapitulate human infection and (2) the difficulty of human sampling, as it is not usually clear where the virus was transmitted. Further, mice and humans mount a potent humoral response against flavivirus infection, and neutralizing antibodies can prevent infection in animal models [105,106]. Therefore, knowledge of T cell behavior in the skin during flavivirus infection is not as detailed as that of the antibody response.

ZIKV and DENV are single-stranded RNA viruses belonging to the *Flaviviridae* family. Both viruses are deposited into the skin after the bite of a mosquito. Keratinocytes are permissive to DENV infection [107]. Similarly, ZIKV has been demonstrated to infect and replicate in epidermal keratinocytes and primary fibroblasts [108]. During flavivirus infection, myeloid cells are recruited in large numbers into the skin at the site of infection; these cells can then serve as new hosts to amplify viral production [109,110]. Langerhans cells and DCs can also become infected by DENV and ZIKV in human skin [111,112,113]. Circulating monocytes have been considered as the primary target for ZIKV replication and may contribute to its dissemination [113,114,115]. A recent study highlighted the importance of IL-27 in the skin for the protection against ZIKV [116]. IL-27 provides an IFN-independent pathway to enhance immune responses through the induction of IL-27RA and STAT1/IRF3 signaling. In addition, the IL-27 receptor subunit alpha (IL27RA) is highly expressed on T cells, and IL-27 signaling can enhance the cytotoxic T lymphocyte numbers. Accordingly, the loss of *Il27ra* leads to a significant increase in morbidity and mortality after cutaneous ZIKV infection [116].

Another unique factor of arbovirus skin infection is the presence of mosquito saliva, which can modulate immune responses to facilitate viral transmission [117]. Although this topic is complex and has been reviewed elsewhere [118,119,120], a few fundamental studies are worth noting to place arbovirus skin infection in a broader context. Mosquito saliva contains several immune and vascular modulators, including vasodilators, anticoagulants, and anti-hemostatic components [119,121]. In non-human primate models of flavivirus infection, mosquito inoculation of DENV or ZIKV results in increased viremia compared to needle-based inoculation [122,123]. In both human and animal models, mosquito saliva increases the recruitment of neutrophils, DCs, M2 macrophages, eosinophils, and mast cells [124]. Mosquito salivary proteins also dampen pro-inflammatory Th1 cytokines such as IFN-γ and IL-2, shifting to Th2-associated cytokines, including the upregulation of IL-10, IL-4, and IL-13 [124]. Recently, the mosquito salivary protein LTRIN was shown to bind to the lymphotoxin-β receptor [125]. This binding inhibits crosstalk between epithelial and immune cells to suppress the cutaneous response to ZIKV infection. Antibody-based blockade of LTRIN in mice leads to decreased ZIKV infection. Mouse models that add mosquito saliva to the initial viral inoculum should provide more information about the impact of saliva on infection and immunity during flavivirus infection. Although mosquito-based viral delivery to mice is challenging, this mode of infection would undoubtedly increase our knowledge of the antiviral response that occurs in humans.

Ticks are also vectors for flavivirus transmission. Following the bite of an infected tick, tick-borne encephalitis virus (TBEV) infection is generally asymptomatic but can also result in encephalitis and severe neurological sequelae in humans. TBEV induces a rapid innate immune response in the skin mediated by type I IFN signaling [126]. Viral interference with host innate intracellular antiviral immunity (such as viral sensing and IFN production) may impair DC maturation and reduce antigen presentation [127]. Many TBEV studies have focused on the innate immune response, clinical features of infection, and vaccine-induced responses [128]. The role of T cells in controlling cutaneous TBEV infection has not been extensively investigated. However, TBEV-specific CD8^+^ T cells can be isolated from human peripheral blood after infection [126]. TBEV-specific T cells proliferate extensively and are highly inflammatory. In mouse models, CD8^+^ T cells may contribute to disease progression rather than enhance protection [129]. Lastly, similar to mosquito bites, tick saliva contains proteins that can stimulate the antiviral immune response.

### 4.2. T Cell Response to Flaviviruses

As previously introduced, little is known about T cell behavior in the skin in response to arbovirus infection. However, T cells in human peripheral blood have been characterized during DENV and ZIKV infection. DENV-specific CD8^+^ T cells isolated from dengue patients are highly activated and proliferative, expressing CD38 and Ki-67 (present in dividing cells) [130]. Circulating CD4^+^ and CD8^+^ T cells express the skin-homing receptor CLA, which promotes T cell migration into the skin [130]. In a DENV murine model, the depletion of CD8^+^ T cells resulted in increased viral replication, providing further evidence for CD8^+^ T cell-mediated control of DENV infection. However, mice were infected intravenously in this study, circumventing skin viral replication [131]. To understand the breadth of the CD8^+^ T cell response, studies have mapped DENV epitopes using a peptide-pulsing approach. These studies identified more than 400 epitopes that induce T cell responses, including epitopes from nonstructural viral proteins [132,133]. HLA alleles correlating with DENV disease susceptibility and severity have also been identified [132].

In addition to DENV, T cell responses to ZIKV infection have been heavily investigated in recent years. Using a mouse model of ZIKV infection, Ngono et al. mapped many T cell determinants to viral structural proteins (including the envelope protein) and nonstructural proteins [134,135]. ZIKV-specific CD8^+^ T cells were marked by polyfunctional IFN-γ and TNF production, along with CD107a expression. ZIKV infection of *Cd8a*^−/−^ mice resulted in high mortality, demonstrating that T cells can protect against ZIKV infection [135]. However, CD8^+^ T cells have also been shown to induce paralysis in ZIKV-infected mice [136]. CD4^+^ T cells have also been shown to protect against primary ZIKV infection [134]. Compared to CD8^+^ T cells, however, CD4^+^ T cells recognize a more restricted set of viral epitopes [134]. More studies will be needed to understand T cell function in flavivirus-infected skin comprehensively.

## 5. Papillomaviruses

### 5.1. Cutaneous Papillomavirus Infection

Papillomaviruses are species-specific, small, non-enveloped DNA viruses that infect the skin and mucosa. There are more than 200 human papillomavirus (HPV) types divided into five phylogenetic genera of alpha, beta, gamma, mu, and nu based on the nucleotide sequences of capsid protein L1 [137]. Many HPVs are commensal and do not cause clinical symptoms in immunocompetent hosts [138]. However, HPV disease prevalence is much higher in patients with immunodeficiencies or immunosuppressed organ transplant recipients [139]. Among the five genera of HPVs, alpha HPVs are tropic for the mucosal epithelium. A subset of alpha HPVs includes high-risk HPV types, such as HPV-16, that are the etiological agents for cervical, anogenital, and oropharyngeal cancers [140,141]. Beta, gamma, mu, and nu genera are skin commensals and may cause benign lesions or warts [142]. Despite the asymptomatic presentation of beta HPV colonization, these viruses can contribute to the development of cutaneous squamous cell carcinoma, especially in association with exposure to ultraviolet (UV) radiation [143].

Cutaneous HPVs preferentially infect the basal keratinocytes, reaching this deep layer of the epidermis via hair follicles or micro-fissures in the skin. HPVs establish a reservoir of persistent infection in undifferentiated basal keratinocytes [144,145,146]. Mature virions are released in the superficial layers of the skin during desquamation [147]. Like high-risk alpha HPVs, oncogenes E6 and E7 of the beta HPVs have potential carcinogenic activities [148,149]. E6 and E7 proteins can prevent host DNA repair, resulting in UV-induced apoptosis, increase telomerase activity, and alter G1 cell cycle progression by binding to tumor suppressor Rb protein [148,150,151]. Thus, although beta HPVs may not directly cause skin cancers, the virus can contribute to cancer initiation in combination with environmental factors such as UV radiation.

Since papillomaviruses are highly species-specific, mouse models to study HPV pathogenesis after natural viral infection have been challenging to develop. Recently, a mouse papillomavirus, *Mus musculus* papillomavirus (MmuPV1), was identified [152]. MmuPV1 can infect both the skin and mucosa of mice, resulting in papillomas similar to those caused by HPV infection [153,154]. After cutaneous MmuPV1 infection, UV radiation further induces papilloma formation and progression to SCC [155]. Interestingly, MmuPV1 E6 and E7 viral proteins retain oncogenic properties mirroring those of the same proteins in HPVs [152]. Early studies with MmuPV1 were carried out in immunocompromised mice [156]. However, later studies used immunocompetent strains, and mice still developed cutaneous papillomas after UV exposure [157]. Thus, although more work needs to be done with this relatively new mouse model of papillomavirus infection, MmuPV1 mouse infection may offer valuable insight into papillomavirus disease control and progression.

### 5.2. T Cell Responses to Papillomavirus Infection

Human and mouse studies of papillomavirus infection have pointed to the importance of the host immune response in maintaining homeostasis and preventing tumor formation after infection, as immunodeficiencies lead to severe HPV disease. Patients with mutations in *GATA2* [158] or *DOCK8* [159], CD28 deficiency [160], and idiopathic CD4 lymphopenia [161] fail to control HPV infection and can experience severe dermatological manifestations of HPV-induced diseases.

Due to the commensal nature of beta HPVs, dissecting the specific mechanisms of T cell-mediated immunity to cutaneous HPV infection has been challenging. HPV viral proteins E5, E6, and E7 have been shown to interfere with the type 1 IFN response to delay APC recruitment and reduce antigen presentation [162,163,164]. HPV-mediated disruption of the initial innate response is thought to dampen the subsequent adaptive immune response. Nonetheless, effector T cells have been shown to target early viral proteins and eliminate HPV-16-infected cells at lesion sites [165,166]. During MmuPV1 infection, antibody-based depletion or genetic deletion of CD4^+^, CD8^+^, and CD3^+^ T cells significantly increases papilloma formation and SCC development, indicating that T cells can restrict papillomavirus-induced disease progression [167]. The adoptive transfer of MmuPV1-experienced splenocytes further reduces papilloma formation [167]. The high prevalence of HPV-associated cancers warrants more investigation into the mechanisms of T cell-mediated control of cutaneous HPV lesions. 

## 6. Conclusions and Perspectives

Despite being a highly effective barrier against pathogens, the skin is also a dynamic immune environment facilitating T cell-mediated immune responses. With progressing technologies, such as time-lapse imaging of T cell movement and function within the skin, recent studies have expanded our understanding of T cell surveillance of viral infections. Some commonalities of T cell priming, activation, traffic, cytotoxic function, and T_RM_ formation have emerged through the aforementioned studies with a diverse range of cutaneous viral infections. For example, we now know that permanent tissue-resident and infiltrating T cell functions are critically dependent on the cellular organization and nature of infected cells, in addition to the expression of cognate antigen in the skin environment. However, we are just beginning to explore the impact of other factors that could move mouse models of cutaneous viral infection closer to conditions encountered during human disease. For instance, the effect of the commensal microbiome or virome on pathogenic cutaneous viral infections has yet to be extensively investigated. Additionally, although ex vivo analyses of T cells from digested tissue have greatly expanded our knowledge of T cell biology in the tissue, these cells are notoriously difficult to remove without impairing their function or survival [168]. Therefore, we should adapt our studies to account for the tissue environment to better understand T cell effector function as it naturally occurs. Finally, viral delivery through insect bites needs to be better modeled to match human encounters in order to fully decipher disease progression and the cutaneous antiviral T cell response [169]. Although we still have much to learn about T cell biology during viral skin infection, the studies reviewed here have significantly enhanced our knowledge of this critical component of antiviral protection. Continuing investigation of T cell function in the skin should yield exciting breakthroughs in T cell-based treatments for a host of human maladies.
